# Label-free optical quantification of structural alterations in Alzheimer’s disease

**DOI:** 10.1038/srep31034

**Published:** 2016-08-03

**Authors:** Moosung Lee, Eeksung Lee, JaeHwang Jung, Hyeonseung Yu, Kyoohyun Kim, Jonghee Yoon, Shinhwa Lee, Yong Jeong, YongKeun Park

**Affiliations:** 1 Department of Physics, Korea Advanced Institute of Science and Technology, Daejeon, 34141 South Korea; 2 KI for Health Science and Technology, Korea Advanced Institute of Science and Technology, Daejeon 34141, Republic of Korea; 3 Graduate School of Medical Science and Engineering, Korea Advanced Institute of Science and Technology, Daejeon 34141, South Korea; 4 Department of Bio and Brain Engineering, Korea Advanced Institute of Science and Technology, Daejeon 34141, South Korea; 5 Department of Biological Sciences, Korea Advanced Institute of Science and Technology, Daejeon 34141, South Korea; 6 TOMOCUBE, Inc., Daejeon 34051, Republic of Korea

## Abstract

We present a wide-field quantitative label-free imaging of mouse brain tissue slices with sub-micrometre resolution, employing holographic microscopy and an automated scanning platform. From the measured light field images, scattering coefficients and anisotropies are quantitatively retrieved by using the modified the scattering-phase theorem, which enables access to structural information about brain tissues. As a proof of principle, we demonstrate that these scattering parameters enable us to quantitatively address structural alteration in the brain tissues of mice with Alzheimer’s disease.

Imaging brain tissues is an essential tool in neuroscience because understanding brain structure provides rich information about brain functions and alterations associated with diseases. Conventional imaging techniques include magnetic resonance imaging (MRI) and positron emission tomography (PET), but such techniques are limited by low spatial resolution (around 100 μm)^1^. To assess detailed structural information and to determine specific sites for assured diagnosis, histology has complemented MRI and PET. The traditional histological method provides the structural information about biological tissues to cellular scales, but relies on labour-intensive staining procedures and provides only qualitative information.

As a complementary method, label-free imaging techniques have been employed in brain imaging. For example, second harmonic generation was used for non-invasive brain imaging in cellular levels^2^. Raman scattering was also applied to image myelin fibres and amyloid plaques^3^ in brains. Optical coherence microscopy has also been utilised for label-free histology of a brain tissue^4^. However, they only provide limited qualitative information about tissue structures. Recently, quantitative phase imaging (QPI) techniques have been utilised for imaging pathological tissue slices^5^,^6^. From measured optical fields, mass density and spatial alterations in biological tissues could be quantified by retrieving refractive index (RI) distribution^7^,^8^,^9^. Because measured optical fields images of tissues with QPI techniques can provide information about scattering properties of tissue structures^10^,^11^, various studies have shown potentials of QPI techniques for label-free tissue imaging^12^ and capability of assessing and diagnosing breast and prostate cancers^13^,^14^.

Here, we present a quantitative label-free approach for the investigation of brain tissue structures. Employing QPI techniques equipped with a motorised stage, holographic images (amplitude and phase delay maps) of mouse brain tissue slices are measured with sub-micrometre resolution. This allows us to perform multi-scale imaging of a whole mouse brain tissue slice, which covers the sub-micrometre scale (subcellular organelles) to the millimetre scale (histological anatomy). We also present a modified version of the scattering-phase theorem^11^, in order to precisely retrieve scattering coefficients (*μ*_*s*_) and anisotropies (*g*) maps of tissue slices from the measured holograms, which allows us to investigate the structural organisations of tissues quantitatively. As a proof of principle, we demonstrate that the scattering parameters of brain tissues of mice with Alzheimer’s disease (AD) are significantly modified, suggesting that the present approach provides a unique means to investigate pathophysiology of neurological disorders.

## Results

**Figure 1:**
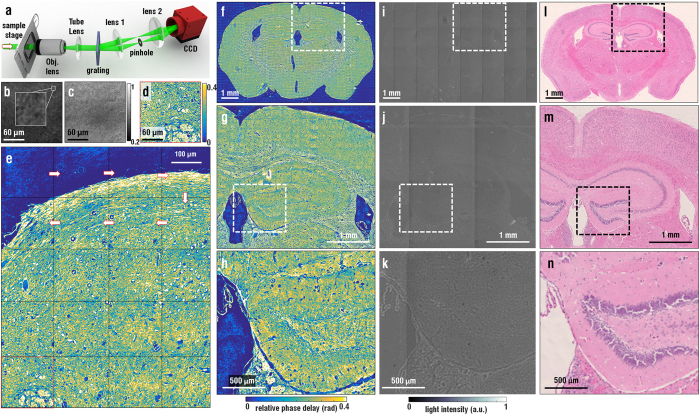
Quantitative phase imaging of a mouse brain tissue slice. (**a**) Diffraction phase microscopy equipped with a translational stage for measuring optical phase delays. (**b**) A representative hologram. (**c**) The retrieved amplitude and (**d**) phase image retrieved from the hologram in (**b**). (**e**) Wide-field phase images of the mouse brain slice, stitched from individual holograms (dotted boxes). Arrows indicate a recording order. (**f–h**) Phase delay image measured with DPM. (**i–k**) Bright-field image of an unstained brain tissue (**l–n**) H&E stained brain tissue slice micrograph of the same brain tissue. For each imaging modality, the magnified image of a selected region in the dashed square is represented below.

The schematic of the QPI setup is shown in Fig. 1a. To perform multi-scale QPI, diffraction phase microscopy (DPM) equipped with a motorised sample stage is utilised (see *Methods*). Employing common-path interferometry, DPM measures optical field images of transparent biological samples with high precision and stability^15^,^16^. To measure wide-field QPI images of a mouse brain slice, segmented QPI images are measured with the sub-micrometre resolution and digitally stitched to generate the wide-field image of the brain tissue slice.

The reconstructed image of a representative brain tissue is presented in Fig. 1f–h. The total field of view is 8.9 × 6.6 mm (horizontal × vertical) and the lateral resolution is 0.8 μm, which is limited only by the numerical aperture (NA) of DPM in this study. The corresponding bright-field images are also presented (Fig. 1i–k). With high contrast and transverse resolution, the phase images quantify the RI of the tissue with respect to the mounting solution (RI = 1.355, see *Methods*) and show the general anatomical features in a brain and spatial variations at subcellular structure level, these being undistinguishable with bright-field imaging. The images of the adjacent slice stained with hematoxylin and eosin (H&E) are also presented in Fig. 1l–n. A side-by-side comparison reveals the matching of general morphological features between the phase image and the conventional histology.

**Figure 2:**
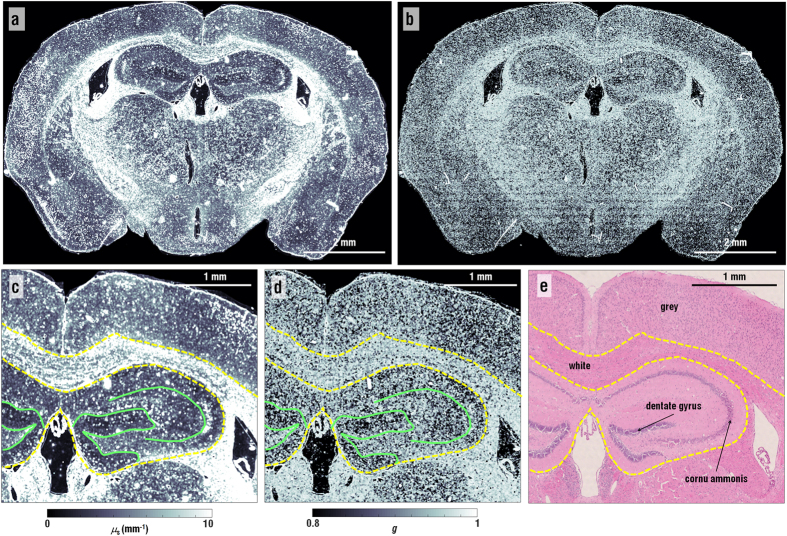
Scattering parameter maps of the brain tissue slice obtained from the quantitative phase image. (**a**) Full-field maps of scattering coefficient and (**b**) anisotropy obtained from the quantitative phase images with the modified scattering-phase theorem. (**c**) Magnified images of scattering coefficient map and (**d**) anisotropy map. (**e**) A matched H&E stained image with (**c**,**d**). Boundaries between grey matter, white matter, and hippocampi are indicated with the dotted yellow lines. Boundaries of dentate gyri and cornu ammonis are presented with the cyan lines

From the measured field images, the maps of *μ*_*s*_ and *g* are precisely retrieved. According to the light scattering theory, *μ*_*s*_and *g*, which are thickness-independent parameters, quantify the attenuation of unscattered intensity per unit distance and the degree of forward scattering, respectively^17^. For a thin biological tissue, *μ*_*s*_and *g* are related to the fluctuation of the scattered field, including both amplitude and phase delay. Recently, the scattering-phase theorem was introduced to retrieve *μ*_*s*_and *g* from quantitative phase images^11^. To consider the alteration in amplitude in addition to the phase of the optical field that transmitted the tissue slices, we propose a modified version of the scattering-phase theorem that measures accurate values of *μ*_*s*_and *g,* as shown in Fig. 2a,b (see *Methods*). The scattering parameters are ill-defined in a ventricular structure because the transmitted field is close to unity. In a sample region, however, the modified scattering-phase theorem precisely reflects the relation between spatial fluctuations of fields and scattering features. The accuracy of the proposed method is validated by measuring the scattering parameters of a known scattering phantom, indicating that phase object approximation underestimates both *μ*_*s*_and *g* (see Supplementary Information). This implies that scattering parameters are sensitive to both amplitude and phase fluctuations due to light scattering, even in the cases of phase objects.

The magnified view around hippocampi and dentate gyri is shown in Fig. 2c,d with the H&E stained micrograph of the adjacent tissue slice for comparison purpose (Fig. 2e).The maps of scattering parameters (*μ*_*s*_ and *g*) provide structural distinctions, thus serving as label-free biomarkers. The boundaries between the grey, the white matter, and the hippocampus formation are clearly separated. Other brain regions such as the thalamus and hypothalamus also exhibit distinct boundaries (the yellow dotted lines in Fig. 2c–e). Importantly, dentate gyri are clearly distinguished from the cornu ammonis (CA) due to their high values of *μ*_*s*_and *g* (the cyan lines in Fig. 2c–e).

The distinct distribution of the values *μ*_*s*_and *g* in the brain regions may relate to the different arrangements of cell types and subcellular compositions in the sub-regions of brains. The area with the highest values of *μ*_*s*_corresponds to white matter. This is consistent with the fact that there are more lipid contents in white matter than in grey matter and hippocampi^18^. The lipid contents cause high local contrast of refractive index (RI), resulting in high values of *μ*_*s*_. The dentate gyri exhibit higher scattering coefficients than the other regions in the hippocampi, primarily due to the presence of dense layers of neurons such as granule cells^19^.

Over the brain tissues, the retrieved map of *g* exhibits a value range between 0.9 to 1, which is comparable to other types of tissues^17^. Nevertheless, the distribution of *g* differs in various sub-regions; the larger size of scattering particles results in the increase of *g*. The map of *g* indicates higher values in the white matter, indicating the tendency of forward-directed light scattering. Together with the retrieved values of *μ*_*s*_, this result indicates that the white matter consists of tissue components of inhomogeneous and large scattering particles, mostly bulky myelin sheaths. The areas of grey matter and hippocampi exhibit lower values of *μ*_*s*_ and *g*, implying that these areas are composed of tissue components of uniformly packed small scattering particles, mostly neuronal cells.

**Figure 3:**
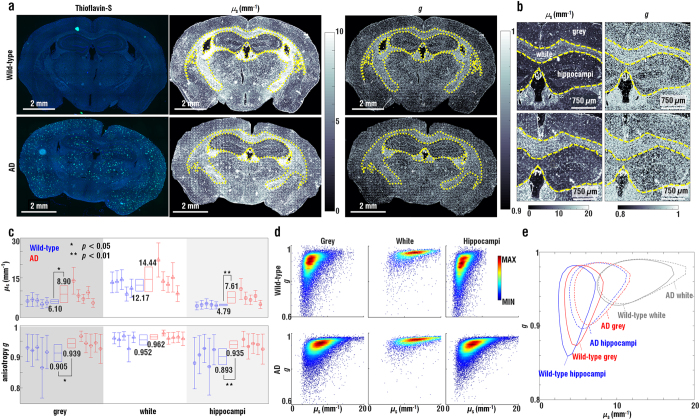
Quantitative analysis of scattering parameters in brain tissues and their alterations in Alzheimer’s disease model mice. (**a**) Full-field thioflavin-S stained fluorescence images and maps of scattering coefficient and anisotropy of representative AD and wild-type mouse. (**b**) Magnified images of scattering parameter maps in (**a**), showing clear distinction between grey matter, white matter, and hippocampi. (**c**) Distributions of scattering coefficients and anisotropy values in grey matter, white matter, and hippocampi regions for five mice from each model. Linear bar: distribution of scattering parameters in each different tissue. Rectangular bar: Sample-mean distribution of the scattering parameters in each sub-region. Range of the bar is mean ± standard deviation. *p < 0.05, **p < 0.01. (**d**) Probability density maps of the scattering parameters in grey matter, white matter, and hippocampi for healthy and Alzheimer’s disease mice. (**e**) 50% density contour plots of the six scatterplots in (**d**).

To demonstrate the applicability of the present approach, we have systematically compared the brain tissue slices of AD model mice with their wild-type littermates. The fluorescent images show that the amyloid plaques stained with Thioflavin-S were deposited only in the brain tissue of AD models (Fig. 3a). We utilised the maps of *μ*_*s*_and *g* for addressing structural alterations in brain tissues associated with AD. Brain tissue slices of the same anatomical regions were measured in five mice from each group. Figure 3a,b show the representative maps for AD and wild-type mice, which show that both the values of *μ*_*s*_and *g* generally increase throughout grey matter and hippocampi in the AD model.

For a quantitative analysis, we statistically analysed the distributions of *μ*_*s*_and *g* in three distinct regions in the brain: grey, white matter, and hippocampi, as shown in Fig. 3c. (Numerical values are shown in Supplementary Tables 2 and 3). A comparison of sample-mean distribution indicates significant increases of *μ*_*s*_and *g* in the grey and hippocampal regions of the AD models, suggesting that AD pathology alters tissues in these areas to exhibit higher RI inhomogeneity and increased size of scattering particles. These results are consistent with the fact that AD is characterised by the accumulation of extracellular amyloid and intracellular neurofibrillary tangles with larger size than granule cells^20^, as well as neuronal cell losses throughout grey matter and hippocampi^21^. The structural changes by AD pathology are mostly distributed over the grey matter and hippocampi, while white matter is relatively spared.

To further investigate the structural alterations in the brain tissues associated with AD, correlative analysis about scattering parameters was performed (Fig. 3d). Figure 3d separately shows the scatterplots of *μ*_*s*_and *g* for the grey matter, white matter and hippocampi in each group. While the scatterplots exhibit a similar correlation of *μ*_*s*_and *g* in white matters for the AD and wild-type mice, they showed a significant difference in the grey matter and hippocampi between the AD and wild-type mice. These trends are clearly visualised when the 50% density boundaries of the scatterplots of *μ*_*s*_and *g* were simultaneously displayed for the six different populations (Fig. 3e).

## Discussion

We present the wide-field label-free QPI of mouse brain tissue slices. We demonstrate the multi-scale phase images of the brain tissues with an image-stitching scheme and the accurate retrieval of the scattering parameters with the modified scattering-phase theorem. Recently, several techniques have been developed to convert existing optical microscopes into QPI instruments^22^,^23^,^24^, which may allow the present method easily accessible to histological laboratories. With AD model mice, we suggest that this approach can be used to systematically quantify the structural changes of AD pathology by measuring quantitative phase images of brain tissue slices. The retrieval of scattering parameters eliminates staining procedures and also provides the quantitative information of the morphology that H&E histology cannot.

The present approach is different from previous reports which utilise the distribution of RI values in tissues^7^,^8^. First, the retrieved scattering parameters in this study are independent of the thicknesses of tissue slides. In order to measure the RI values of tissues, one should assume the axial homogeneity of RI in a tissue or need to use a complicated tomographic instrument^5^,^25^. The present method utilises the measured 2-D quantitative phase images to retrieve scattering parameters, which can avoid these technical difficulties and enables to easily access pathologically relevant information. Second, the present approach enables the spatially resolved information. Therefore, the retrieved optical scattering parameters can be readily compared and complimented to the existing histological methods in order to address anatomical information of tissue slices.

Possible errors in our experiments can be resulted by remaining field-dependent aberrations, speckle noises or the deformation of biological tissues during paraffin embedding process^26^. This can be resolved if we improve the image reconstruction algorithm, employ incoherent light source for low-coherence interferometry^9^,^22^, or compensate for the deformation errors^27^. Nevertheless, we found that the modified scattering-phase theorem and the current experimental implementation provide scattering parameters of brain tissues, in good agreement with previously reported results^28^.

Although this work has focused on brain tissue slices, the approach is sufficiently broad and general, and it will directly offer novel approaches for general histopathology. The present method has potentials to be applied to investigate prognosis of various neurodegenerative diseases such as Parkinson’s disease. In order to further extend the present method, hyperspectral^29^,^30^,^31^ or polarization-sensitive^32^,^33^ QPI techniques can also be exploited. Recently, several quantitative phase microscopes have been commercially available^34^,^35^, which make this approach readily available to many researchers. We anticipate that extension of this work will directly offer novel approaches for comprehensive histopathology.

## Materials and Methods

### Optical setup of diffraction phase microscopy

In order to obtain the optical field images of brain tissue slices, DPM has been employed. DPM is a common-path interferometric microscopy, capable of quantitative phase imaging of biological samples with high resolution and phase stability^15^. As an illumination source, a diode-pumped solid-state laser (*λ* = 520 nm, LP520-SF15, Thorlabs Inc., USA) is employed. An inverted microscope (IX71, Olympus American Inc., USA) is equipped with a motorised *xy*-scanning stage (MLS203, Thorlabs Inc., USA) and a 10× objective lens (0.4 NA, UPlanSApo 10×, Olympus American Inc., USA). Via the objective and a tube lens, the optical field of a sample is imaged onto the image plane. To acquire a hologram, a grating (92 grooves/mm, 46-071, Edmund Optics Inc., USA) is placed at the sample plane, and a customised pinhole filter is located on the Fourier plane. The diffraction grating splits the sample field into many diffraction orders. Among them, only two orders (the 0th and the 1st diffraction order) of the diffracted beams are further projected onto a CCD plane via a 4-*f* telescopic imaging system. The 1st diffraction beam is spatially filtered using the pinhole (25 μm diameter) located at the Fourier plane, which serves as a reference beam.

Both the sample and the reference beams interfere at the CCD plane, generating a spatially modulated hologram (Fig. 1b). A total magnification of the imaging system is 71×. Individual holograms were recorded by a sCMOS camera (C11440-22C, Hamamatsu Inc., Japan). Using a field retrieval algorithm^36^, the amplitude and the phase delay maps of a sample are retrieved (Fig. 1c,d). The amplitude image (Fig. 1c) does not exhibit enough contrast to distinguish geometric anatomy and cellular components due to the optical transparency of tissues. The phase image of the tissue (Fig. 1d), however, clearly shows the meso-scale and the sub-micrometre scale structures of the tissue with significant phase imaging contrast. The resolution of the imaging system is 0.8 μm, which is limited by the numerical aperture of the objective lens (NA = 0.4).

### Stitching segmented quantitative phase images

The full-field phase image of a mouse brain tissue is reconstructed from the measured segmented phase images by stitching the segmented images. In order to achieve wide-field QPI, the motorised stage laterally translates a tissue slice in a fully automated manner. The field of view of a single mosaic image is 187 × 187 μm, which overlaps 10% with adjacent images. A custom-made MATLAB code was used to reconstruct a full-field phase image from more than 1000 mosaics, i.e. 1908 mosaics for Fig. 1f (53 × 36).

The reconstruction includes several imaging processes in order to correctly stitch individual segmented phase images. First, the static phase distortion due to the aberration of the imaging system is removed by subtracting mosaic images by the background phase image. Remaining phase error was reduced by matching the overlapped adjacent areas between mosaic images. A constant phase value is added to the reconstructed image so that the background area has the phase value of zero. Both amplitude and phase images are corrected similarly.

### Sample preparation

For the preparation of brain tissue slices, we used 22-month-old male AβPP^SWE^/PS1ΔE9 transgenic mice (Tg) as AD models and their wild-type (Wt) littermates as control models. The mice were deeply anaesthetised by intraperitoneal injection of ketamine/xylazine and perfused transcardially with phosphate buffered saline (PBS). The brains were postfixed in 4% paraformaldehyde at 4 °C overnight, and dehydrated through a series of increasing gradient of ethanol using automatic tissue processor (TP1020, Leica). The dehydrated brain tissues were embedded in paraffin block and sliced into 3–3.5 μm-thick coronal sections using microtome (RM2245, Leica Microsystems, Germany). <>**r** After deparaffinization using xylene and rehydration in a decreasing gradient of ethanol, Thioflavin-S and hematoxylin and eosin (H&E) stains were used to observe deposition of amyloid plaques and anatomical structures. The tissue sections were mounted with Histomount (National Diagnostics Inc., USA) and cover-slipped. The sample preparation procedures and the methods were reviewed and approved by the Institutional Review Board (KA-2015-03). All the experiments in this study were carried out in accordance with the approved guidelines.

### Derivation of modified scattering-phase theorem for accurate retrieval of *μ*_*s*_and *g*

The scattering-phase theorem is a recently proposed method which retrieves maps of *μ*_*s*_ and *g* from a spatial fluctuation of RI^11^. However, this method computes the values of *μ*_*s*_and *g* by assuming uniform amplitude distribution of a sample. In order to improve the accuracy of the retrieved scattering parameters, we modified the original scattering-phase theorem to consider amplitude fluctuation as well as phase fluctuation in a sample. The detailed algorithm of the modified scattering-phase theorem is explained below.

By Beer-Lambert law, *μ*_*s*_ is accurately retrieved as,


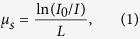


where 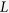 is the thickness of a tissue, 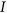 the unscattered light intensity, and 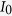 the total illumination intensity, which is the sum of scattered intensity and unscattered intensity. Here, *I* and *I*_*0*_ are retrieved by Fourier Transform Light Scattering (FTLS) technique^37^,^38^. FTLS is a numerical method to obtain the angular light scattering map in the far-field by numerically propagating the measured 2-D optical field of a sample at the sample plane, *U*(**r**) = *A*(**r**)exp[*iϕ*(**r**)], where *A(***r**) and *ϕ*(**r**) are the amplitude and the phase of the field. Then, the 2D angular light intensity in the far-field has the following relation,


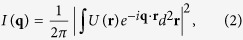


where **q** is the spatial frequency vector. By setting **q** = 0, *I* is calculated, and then *μ*_*s*_can be directly computed from Eq. (1). Unlike the original scattering-phase theorem, both amplitude and phase are considered in calculating *μ*_*s*_, without setting *A*(**r**) = 1.

Anisotropy coefficient *g* is defined as the average cosine of the scattered angle after a single scattering process as,


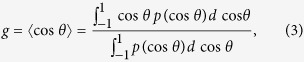


where *p*(cos *θ*) is the intensity distribution of the angular scattering plot. Since the scattering angle is related with the spatial frequency (|**q**| = 2*k*_*0*_sin *θ*), Eq. (3) can be rewritten as follows:


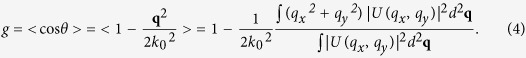


Applying Parseval’s theorem followed by differentiation theorem, the following relationship is obtained:


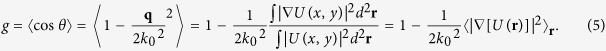


According to the definition, anisotropy should be computed where the thickness of a tissue is equivalent to the mean free path (*l*_s_ = 1/*μ*_s_). Therefore, we numerically propagate the field through *N* = *l*_*s*_*/L* layers. Here, we compute *μ*_s_ from the method in the previous section. The result of the numerical propagation of Eq. (5) is as follows:


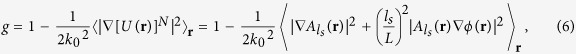


where *A*_*ls*_(**r**) = *A*(**r**)^*N*^ and <>**r** denotes spatial averaging over the sub-region window. For the results analysed in this work, the sub-region window was set to be 18 μm × 18 μm, which is large enough to represent local structural variations and small enough to correspond to cellular sizes.

In the original version of the scattering-phase theorem^11^, the uniform amplitude assumption yields the following result:


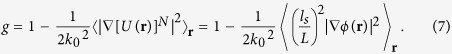


However, in our modified scattering-phase theorem, the Eq. (6) is directly used for the accurate calculation of the anisotropy.

### Statistical Analysis

We utilised MATLAB in order to calculate *P* values by Mann-Whitney-Wilcoxon rank tests for comparing the sample means of scattering coefficients and anisotropy values between normal and AD models. All of the numbers following the ± sign in the text are standard deviations.

## Additional Information

**How to cite this article**: Lee, M. *et al.* Label-free optical quantification of structural alterations in Alzheimer’s disease. *Sci. Rep.* **6**, 31034; doi: 10.1038/srep31034 (2016).

## Supplementary Material

Supplementary Information
