# Supplementary material for: Label-free optical quantification of structural alterations in Alzheimer’s disease
Source: Sci Rep. 2016 Aug 3;6:31034. doi: 10.1038/srep31034 (PMC4971571; doi:10.1038/srep31034)
Supplement: Supplementary Information [file srep31034-s1.pdf]

Label-free optical quantification of structural alterations in Alzheimer's disease

Moosung Lee[a,+], Eeksung Lee[b,d,f+], JaeHwang Jung[a], Hyeonseung Yu[a], Kyoohyun Kim[a], Jonghee Yoon[a], Shinhwa Lee[c], Yong Jeong[d,*], YongKeun Park[a,e*]

[a] Department of Physics, Korea Advanced Institute of Science and Technology, Daejeon, South Korea, 34141

[b] Graduate School of Medical Science and Engineering, Korea Advanced Institute of Science and Technology, Daejeon 34141, South Korea

[c] Department of Biological Sciences, Korea Advanced Institute of Science and Technology, Daejeon 34141, South Korea

[d] Department of Bio and Brain Engineering, Korea Advanced Institute of Science and Technology, Daejeon 34141, South Korea

[e] TOMOCUBE, Inc., Daejeon 34051, Republic of Korea

[f] Current affiliation : Department of Neurology, Seoul National University Bundang Hospital, 82, Gumi-ro 173 Beon-gil, Bundang-gu, Seongnam-si, Gyeonggi-do 13620, Republic of Korea

+these authors contribute equally to this work.

*corresponding authors: Y.K.P (yk.park@kaist.ac.kr); Y.J (yong@kaist.ac.kr)

Supplementary figures and text:

Supplementary Figure 1 Images of the scattering phantom.

Supplementary Table 1. Comparison of computing methods for a scattering phantom.

Supplementary Table 2. Sample mean distributions of scattering parameters in brain tissues and their alterations in Alzheimer's disease model mice.

Supplementary Table 3. Individual distributions of scattering parameters in brain tissues.

Supplementary Text: The validation of the modified scattering phase theorem

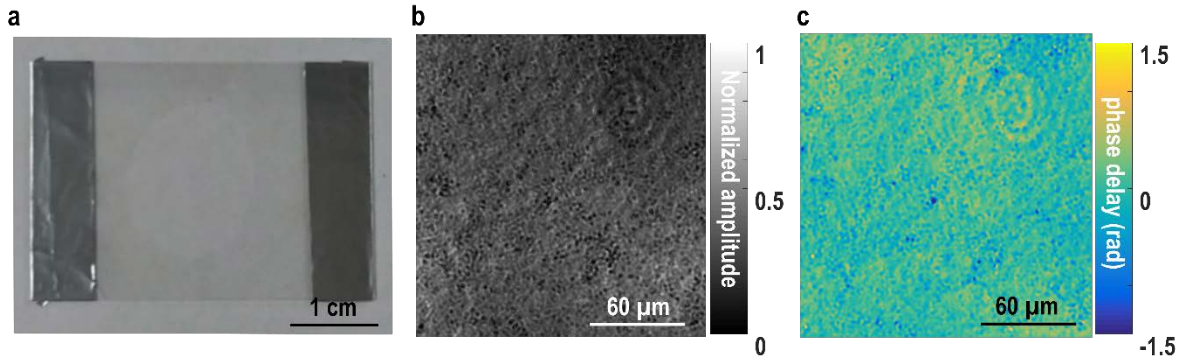

Supplementary figure 1. Images of the scattering phantom. **(a)** A scattering phantom made with agarose gel and polystyrene beads. **(b)** An amplitude image of the bead scattering phantom. **(c)** A phase image of the bead scattering phantom**.**

Computing method	Scattering coefficient (mm^{-1})	Anisotropy g
Numerical simulation	4.772	0.977
Scattering-phase theorem (assume uniform amplitude)	2.053±0.916	0.911±0.036
modified scattering-phase theorem	4.358±1.335	0.969±0.042

Supplementary table 1. Comparison of computing methods for a scattering phantom.

Sample means	Scattering coefficient (mm^{-1})	Anisotropy g
White matter – healthy	12.17 ± 2.19	0.952 ± 0.018
White matter – AD	14.44 ± 4.47	0.962 ± 0.008
Grey matter – healthy	6.10 ± 0.76	0.905 ± 0.030
Grey matter – AD	8.90 ± 3.29	0.939 ± 0.016
Hippocampi – healthy	4.79 ± 0.33	0.893 ± 0.029
Hippocampi – AD	7.61 ± 2.16	0.935 ± 0.018

Supplementary table 2. Sample-mean distributions of scattering parameters in brain tissues and their alterations in Alzheimer's disease model mice.

Region	Sample	Scattering coefficient (mm^{-1})	Anisotropy g
**Grey matter**	Healthy 1	6.31 ± 2.18	0.924 ± 0.051
	Healthy 2	6.93 ± 2.62	0.915 ± 0.058
	Healthy 3	6.55 ± 2.25	0.926 ± 0.047
	Healthy 4	5.01 ± 1.61	0.853 ± 0.114
	Healthy 5	5.70 ± 1.99	0.909 ± 0.062
	AD 1	14.17 ± 5.04	0.964 ± 0.019
	AD 2	8.24 ± 2.18	0.940 ± 0.036
	AD 3	7.06 ± 1.71	0.931 ± 0.043
	AD 4	9.50 ± 2.19	0.941 ± 0.035
	AD 5	5.54 ± 1.59	0.920 ± 0.054
**White matter**	Healthy 1	13.17 ± 3.95	0.958 ± 0.021
	Healthy 2	13.68 ± 4.07	0.952 ± 0.026
	Healthy 3	14.05 ± 3.05	0.966 ± 0.025
	Healthy 4	8.83 ± 2.41	0.922 ± 0.050
	Healthy 5	11.11 ± 3.34	0.961 ± 0.019
	AD 1	21.26 ± 6.17	0.975 ± 0.011
	AD 2	13.56 ± 3.39	0.957 ± 0.022
	AD 3	12.52 ± 2.88	0.960 ± 0.019
	AD 4	15.68 ± 5.94	0.962 ± 0.019
	AD 5	9.18 ± 3.08	0.956 ± 0.024
**Hippocampi**	Healthy 1	4.41 ± 1.54	0.870 ± 0.097
	Healthy 2	4.82 ± 1.61	0.899 ± 0.061
	Healthy 3	5.30 ± 1.81	0.922 ± 0.052
	Healthy 4	4.81 ± 1.56	0.857 ± 0.106
	Healthy 5	4.61 ± 1.35	0.917 ± 0.057
	AD 1	10.86 ± 3.84	0.954 ± 0.029
	AD 2	7.35 ± 2.24	0.929 ± 0.046
	AD 3	6.66 ± 1.73	0.939 ± 0.037
	AD 4	8.18 ± 1.97	0.936 ± 0.038
	AD 5	4.99 ± 1.49	0.919 ± 0.056

Supplementary table 3. Individual distributions of scattering parameters in brain tissues.

Supplementary Text: The validation of the modified scattering phase theorem

In this proposed work, the quantitative label-free imaging of mouse whole brain tissue slices assesses structural information through scattering parameters. In order to accurately quantify the scattering coefficient μ_s and the anisotropy g of biological tissues, we introduce a modified version of the scattering-phase theorem[1]. The scattering-phase theorem is a recently proposed method which retrieves maps of μ_s and g from a spatial fluctuation of refractive index (RI). However, this method computes the values of μ_s and g by assuming uniform amplitude distribution. In order to improve the accuracy of the retrieved scattering parameters, we modified the original method to consider amplitude fluctuation in addition to phase fluctuation. In the following sections, we validate the modified scattering-phase theorem by measuring scattering parameters of known tissue phantoms.

a. Scattering phantom preparation

We prepared an 18 μm thick scattering phantom (Fig. S1a) with the mixture of agarose and polystyrene beads. To make a thin phantom mold, we taped two sides of a cover glass with 18-μm-thick aluminum foils. Next, the phantom solution was prepared by making a solution of 1.05% polystyrene bead (89954-5ml-F, Sigma-Aldrich, 0.985 μm diameter) and 2% low-melting agarose gel (A9414, Sigma-Aldrich) in deionised water. After the phantom solution was poured in the mold, the solution was pressed by another coverslip on top until it becomes solid. The thickness of the phantom was equivalent to the thickness of the foil, confirmed by measuring with calipers. The computed scattering length (l_s~ 200 μm) was eleven times longer than the sample thickness, which is a valid condition for the scattering-phase theorem[1].

b. Mie scattering simulation for computing scattering parameters of scattering phantoms

As reference values, we numerically predicted the values of μ_s and g of the scattering phantom using the Mie theory[2]. In the Mie theory, the scattering angle is considered from 0° (forward scattering) to 180° (backscattering), as shown in the following definitions:

$$\mu_s = \rho_s \sigma_s, \quad (1)$$

$$g = \langle \cos\theta \rangle = \frac{\int_{-1}^{1} \cos\theta \, p(\cos\theta) d\cos\theta}{\int_{-1}^{1} p(\cos\theta) d\cos\theta}, \quad (2)$$

where μ_s is the scattering coefficient, ρ_s the number density of dielectric spheres, σ_s the scattering cross section of each sphere, g the anisotropy, and $p(\cos\theta)$ normalised angular scattering intensity. In an actual experiment, however, the microscopy setup only receives the angular light scattering information corresponding to the numerical aperture of the objective lens. Since the 2-D field map is acquired with the limited numerical aperture in QPI, the limited angle coverage should be also considered in the Mie scattering simulation for a fair comparison.

Therefore, we took into account the numerical aperture (NA = 0.4) of an imaging system and computed the scattering parameters obtained from the corresponding scattering angle (θ_{NA} ~ 23.6°). The expected values of μ_s and *g* measured in DPM in this study are expressed as the following equations:

$$\mu_s = \rho_s \sigma_s ' = \rho_s \int_{\cos(\theta_{NA})}^{1} \int_{0}^{2\pi} \frac{d\sigma}{d\Omega} d\phi d\cos\theta, \quad (3)$$

$$g = \langle \cos\theta \rangle = \frac{\int_{\cos(\theta_{NA})}^{1} \cos\theta\, p(\cos\theta) d\cos\theta}{\int_{\cos(\theta_{NA})}^{1} p(\cos\theta) d\cos\theta}, \quad (4)$$

where $d\sigma/d\Omega$ is the differential cross section of the sphere. The expected values are shown in **Supplementary Table 1.**

c. Experimental validation of the modified scattering theorem

Using DPM, we obtained the light field information of the scattering phantom (Fig. S1b-c). We used the field information in order to compute the scattering coefficients and anisotropies of the phantom in the modified method and the original scattering phase theorem. In the computation, the sub-region window was chosen as 18 μm × 18 μm, which was large enough to represent local structural variation. In **Supplementary Table 1**, the first row presents scattering parameters of the phantom using numerical calculation based on the Mie theory[2]. The results of conventional scattering phase theorem and modified computation method are presented in the second and third row, respectively. As expected, the conventional method underestimates both μ_s and *g*, attributed to uniform amplitude assumption. However, the modified method is closer to numerically predicted values because the suggested method considers both amplitude and phase variation of the field. Small errors can be explained by inhomogeneous distributions of the beads, speckle noises or sample deformations. Nevertheless, we found that our modified method is more accurate than the original method. This implies that scattering parameters are sensitive to both amplitude and phase fluctuations due to light scattering, even for phase objects. Therefore, we adopted the modified scattering theorem to compute the scattering parameters over mouse brain tissues.

Supplementary References

1 Wang, Z., Ding, H. F. & Popescu, G. Scattering-phase theorem. *Opt. Lett.* **36**, 1215-1217 (2011).

2 Hulst, H. C. v. d. *Light scattering by small particles*. (Dover Publications, 1981).
